# Uncovering Clinical Features of De Novo Philadelphia Positive Myelodysplasia

**DOI:** 10.1155/2017/5404131

**Published:** 2017-02-21

**Authors:** Aristides Armas, Chen Chen, Martha Mims, Gustavo Rivero

**Affiliations:** ^1^Baylor St. Luke's Medical Center, Houston, TX 77030, USA; ^2^Baylor College of Medicine, Section of Hematology and Oncology, 1 Baylor Plaza, Houston, TX 77030, USA; ^3^Department of Pathology, Baylor College of Medicine, 1 Baylor Plaza, Houston, TX 77030, USA; ^4^The Dan L. Duncan Comprehensive Cancer Center at Baylor College of Medicine, 1 Baylor Plaza, Houston, TX 77030, USA

## Abstract

Myelodysplastic syndrome (MDS) is cytogenetically heterogeneous and retains variable risk for acute myeloid leukemia transformation. Though not yet fully understood, there is an association between genetic abnormalities and defects in gene expression. The functional role for infrequent cytogenetic alteration remains unclear. An uncommon chromosomic abnormality is the presence of the Philadelphia (Ph) chromosome. Here, we report a patient with Ph+ MDS treated with low dose Dasatinib who achieved hematologic response for 7 months. In addition, we also examined the English literature on all de novo Ph + MDS cases between 1996 and 2015 to gain insight into clinical features and outcome.

## 1. Case Description

**Figure 1:**
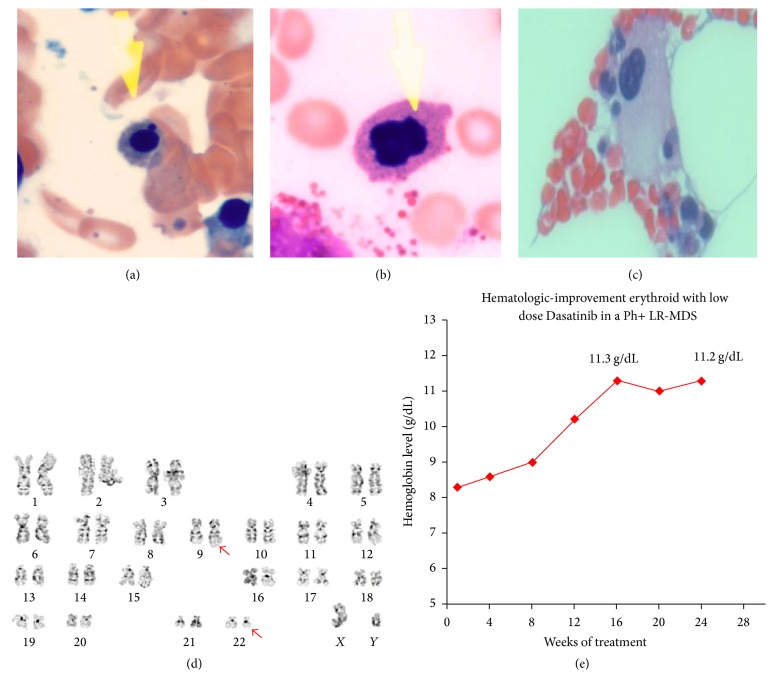
Bone marrow aspirate, biopsy, metaphases cytogenetic, and response to Dasatinib in a Ph+ LR-MDS patient. (a) Erythroid precursor with nuclear irregularity, budding, and nuclear/cytoplasm desynchronization. (b) Dysplastic erythroid precursors with karyorrhexis. (c) shows hypolobated dysplastic megakaryocytes. (d) Patient karyotype showing t (9; 22) translocation. (e) Progressive increase in hemoglobin level for a Ph + LR-MDS [RCMD] patient treated with low-dose Dasatinib. Progressive H-E was observed at 12 weeks. HI-E was sustained at 24 weeks of treatment.

A 74-year-old male was evaluated for refractory anemia. He had a history of gallbladder cancer treated with radiation (RT) in 2010. His Hb, mean corpuscular volume (MCV), and red cell distribution width (RDW) were 8.3 g/dL, 86.1 fL, and 19.8%. White blood cell count, absolute neutrophil count (ANC), and absolute monocyte count (AMC) were 5400/*μ*L, 3800/*μ*L, and 1080/uL. There was no evidence for basophilia or thrombocytosis. His reticulocyte count, erythropoietin (EPO), and vitamin D level were 0.8%, 25.8 MIU/ML (2.6–18.5), and 76 pg/mL. Ferritin level was 120 ng/mL. Physical exam demonstrated no hepatosplenomegaly. Peripheral blood smear showed normocytic, normochromic anemia with scattered target cells. White blood cells were unremarkable and platelets were adequate. His bone marrow was hypocellular with trilineage dysplasia. His erythroid cells showed nuclear irregularities, budding, and nuclear/cytoplasmic desynchronization (Figures 1(a) and 1(b)). Micromegakaryocytes were hypolobated (Figure 1(c)). Immunohistochemistry revealed no evidence of blasts or monocytosis. Bone marrow findings were consistent with refractory cytopenia with multilineage dysplasia (MDS-MLD) [1]. Karyotype was 46, XY, t (9:22) [4], XY [16] (Figure 1(d)). MDS fluorescence in situ hybridization (FISH) including probes for 5q31, 7q31, 8 centromere, 11q23, and 20q12 was negative. Chromosome microarray analysis (CMA) showed no pathogenic copy number variation (CNV) or copy number neutral loss of heterozygosity (LOH). Next generation gene sequencing demonstrated no relevant leukemia mutations in* CSFR1*,* SF3B1*,* SRSF2*,* U2AF1*,* NRAS*,* KRAS*,* FLT3*,* JAK2*,* JAK3*,* DNMT3A*,* KIT*,* PHF6*,* PDGFRA*,* CDKN2A*,* IDH1*,* IDH2*,* TET2*,* EZH2*,* CEBPA*,* EP300*,* PTPN11*,* P53*,* CREBBP*,* IKZF1*,* IKZF3*,* NOTCH1*,* RUNX1*,* WT1*, and* NPM1*. Real time polymerase chain reaction (PCR) for BCR-ABL1 (Major p210 form) was 24.1180%, International Scale (IS). Estimated R-IPSS score was 3 (hemoglobin [8.3 g/dL] = 1; platelets [341000/*μ*L] = 0; absolute neutrophil count [3800/*μ*L] = 0; cytogenetic = 2; blast = [0%] = 0). Trilineage dysplasia, refractory anemia without basophilia, karyotypic, and molecular clonal evidence for Ph + disease prompted treatment with Dasatinib at 20 mg orally daily. A low dose was selected given potential risk for worsening cytopenias. His hemoglobin progressively increased by 3 g/dL after 12 weeks of treatment (Figure 1(e)). Response was maintained for a total of 24 weeks (Figure 1(e)). BCR-ABL1 transcripts were undetectable (0.0006%, IS) after 4 months of treatment. After 7 months of follow-up, patient opted for hospice care after developing pneumonia that led to respiratory failure.

## 2. Methods

In addition to our case, 9 patients diagnosed with de novo Ph+ MDS were identified from PubMed search [2–8]. De novo Ph+ MDS was defined as unequivocal morphologic evidence for myeloid dysplasia and initial karyotype examination positive for Ph abnormality. Given potential biologic confounders, treatment related MDS, CMML, and MDS/MPN Ph+ cases were excluded. In our 10 patients' cohort, we investigated world health organization (WHO) 2008 classification, demographics, histopathologic bone marrow findings, and clinical outcome. Our primary aim was to identify relevant disease features that could facilitate disease management.

## 3. Cohort Analysis

Patient's characteristics and cytogenetic abnormalities are depicted in Table 1. Most of patients were males 6/10 (60%). Median age at diagnosis was 66 years (range, 49–74). According to WHO 2008, 3/7 (43%) and 2/7 (29%) patients were RAEB-2 and RCMD, respectively. RAEB-1 and RCUD (1 each) represented two additional patients with morphologic available data. Median hemoglobin, platelet, white, and blast count were 8.75 g/dL, 88 K/uL, 4.35 K/uL, and 4%. Among 10 patients, Ph translocation, as a sole chromosomic abnormality, was detected in 5/10 (50%). Trisomy 8 occurred commonly as an additional karyotypic alteration in 4/10 (40%) of patients and was frequently associated with complex cytogenetic spanning monosomies 5 and 7. TKI treatment resulted in hematologic remission in 4 patients leading to a median response duration of 7 months. In 5 patients with available follow-up, median time to AML transformation was 7 months. Interestingly, in patients with de novo Ph+ MDS, a platelet count of more or less than 100 K/uL resulted in an overall survival (OS) of 32 versus 9 months.

## 4. Discussion

MDS is cytogenetically heterogeneous presenting with karyotypic abnormalities in about 50% of patients. Chromosomic gains and, most commonly, somatic loss of tumor suppressor genes (TSG) by structural and epiphenomenon's modifications result in impaired differentiation [9]. Translocation (9; 22) is frequently observed in chronic myelogenous leukemia (CML). The 2008 world health organization (WHO) and the 2016 revision for classification of myeloid neoplasm emphasizes that CML BCR-ABL1^+^ phenotype must include significant myeloproliferation. A distinction for chronic myelomonocytic leukemia (CMML) is the prerequisite for persistent monocytosis in peripheral blood ≥ 1000^9^/L [1, 10]. Paradoxically, the translocation (9; 22) is rarely associated with dysplastic hematopoiesis suggesting that the abnormality may have antagonistic disease-initiating role. In this report, we describe a Ph+ MDS patient exhibiting refractory anemia and bone marrow trilineage dysplasia that attained HI-E after low dose Dasatinib. Lack of peripheral blood and bone marrow proliferation favored clonal disorder unrelated to myeloproliferative neoplasm. The presence of Ph+ metaphases and low-grade BCR-ABL transcripts suggest that his MDS was, at least in part, resultant from aberrant kinase overexpression. Given the rare nature of de novo Ph + MDS, we selected 9 additional cases to evaluate disease presentation and outcome. Previously, Keung et al. described three de novo Ph+ MDS cases and reviewed 18 additional patients [4]. However, contrasting with our de novo cohort, his study included Ph + treatment-related disease and CMML cases. In addition, about 48% of patients lacked Ph+ chromosomic abnormality and rather acquired the translocation sequentially at disease progression. It is possible that Keung et al. study design limits our ability to characterize disease initiating mechanisms and de novo disease outcome. We observed that 50% of patients present with t (9; 22) as a sole abnormality. However, typical MDS chromosomic abnormalities, such as +8, frequently coexist in complex Ph+ metaphases. The disease exhibits high-risk features characterized by complex karyotype and elevated proportion of blast and thrombocytopenia. Previous studies have demonstrated that thrombocytopenia adversely impacts MDS outcome [11]. Neukirchen et al. specifically highlights that patients presenting with platelet count lower than 100000/uL retained inferior outcome [12]. It is possible that Ph+ MDS exhibits similar prognostic variables as those observed in Ph negative MDS. A short median time to AML transformation of 7 months suggests that Ph + MDS is a highly aggressive disease, especially if it is associated with complex karyotype and thrombocytopenia. In Keung et al. study [4], median time progression to AML was reported as 13 months. This most favorable prognosis may be biased by inclusion of indolent Ph+ myeloproliferative disorders.

Three Ph+ MDS patients developed isolated refractory anemia, normal platelets, low blast count, and more favorable outcome. This led us to hypothesize that their IE functionally originates from similar Ph negative low risk MDS mechanisms. Given potential BCR-ABL disease initiating role, TKI represents an attractive intervention. However, to date, the efficacy of this therapy on Ph+ MDS remains poorly characterized. In our cohort study, TKI treatment resulted in about 7 months' response in 4 patients. Similarly, TKI was feasible in our reported case demonstrating a correlation between HI-E and molecular remission. Interestingly, low dose of Dasatinib led to limited side effects. IE is associated with abnormal paracrine/autocrine pathways leading to hemopoietic failure; specifically elevated IL-1*β*, TNF-*α*, and INF-*γ* levels are central to low risk-MDS (LR-MDS) [13]. In recent years, the role of deregulated innate immunity has emerged as potential etiology for LR-MDS. Although our patient's HI-E could be attributed to Dasatinib induced BCR-ABL inhibition, it is possible that an “off-target” Dasatinib effect on deregulated cytokines facilitated IE improvement. In vitro studies have demonstrated that Dasatinib suppresses the production of IL-6 and TNF*α* and favors production of anti-inflammatory cytokines, such as IL-10 in primary macrophages [14]. In addition, Src-family kinases (SFK), a potential Dasatinib target, participates in toll-like receptor-2 (TLR-2) mediated NF-kB activation leading to elevated IL-6 and TNF*α* levels [15]. Recent reports link TLR-2 deregulation and MDS pathogenesis [16]. Dasatinib may potentially cooperate to normalize IL-6 and TNF*α* levels inappropriately elevated in LR-MDS.

There are limitations to our study. The retrospective nature of the study could have biased our survival estimation for patients with high and low risk features given inaccurate censoring at the time of publication. Concerning our case, the lack of posttreatment bone marrow evaluation limits our ability to assign a direct favorable TKI effect to improve dysplastic marrow features. However, low dose Dasatinib led to molecular remission and improved IE. This may suggest a favorable drug effect on Ph + MDS disease initiating mechanisms and chronic marrow inflammation. To date, our study is the first to investigate a cohort of newly diagnosed Ph + MDS. The disease potentially recapitulates low and high risk features observed in Ph negative disease. It is likely that high risk Ph+ MDS could exhibit short latency to AML necessitating early chemotherapy and even allo-stem-cell transplantation. Future directions should evaluate the role and potential mechanism of action of TKIs in Ph + MDS patients presenting with low risk features.

## Figures and Tables

**Table 1:** 

Case	Age/sex	WBC^*∗*^	Hemoglobin (g/dL)	Platelets (K/uL)	AMC^*∗∗*^(uL)	Bone marrow blast (%)	WHO^*∗∗∗*^	Cytogenetic	Treatment	Outcome^±^	Time to AML^*∗∗∗∗*^(months)	Ref.
1	49/F	6.5	8.2	425	585	0	MDS	46,XX,t(9;22)(q34;q11)	BSC^∧^	AML	32	[2]
2	70/F	6.4	9.5	316	384	NA	MDS	46,XX[3]/46,XX,t(9q;22q) [12]	NA	Alive at 45 m		[3]
3	74/M (Case)	5.3	8.3	341	1080	0	RCMD^+^	44, XY, t (9:22) [4], 46,XY [16]	TKI	(alive) 7 months after diagnosis		
4	59/M	1.3	9.2	78	78	0	MDS	46,XY,t(9;22)(q34;q11)[20]	TKI^+^ + allo- stem cell transplant	(alive)	4^*ε*^	[4]
5	66/F	0.9	4.4	52	54	2	RCUD^+++^	46,XX,+8,t(9; 22;16)(q34;q11.2;q23)[4]/4 6,XX,idem,der(12)t(12;17) (p11.2;q11.2)[7]/46, XX [9].	Melphalan	(died) Developed skin Granulocytic sarcoma.	9	[4]
6	67/M	2.7	10.4	52	81	4	RCMD	45, XY,+3,-5,-7,-7,- 20,+mar,+mar/45,XY,+3,- 4,-8,-9,-11,-18,-20,-21,- 21,+mar,+mar,+mar,+mar, +mar,+breaks. FISH t(9;22)	TKI	(died) 1 year after diagnosis developed Fungal Pneumonia		[5]
7	62/M	1.8	11.9	3	NA	5	RAEB^*Υ*^-1	45,XY,-5,-7,+8, - 12, - 16,- 22, + marE[?del(11)(q11)], + marC[?der(11)t(?;11;22)], + del(22)(q11)	BSC	(died)	2	[6]
8	67/F	3.4	11.5	111	NA	10	RAEB-2	46 XX, t(9;22)	TKI	(alive)	7	[7]
9	69/M	5.3	8.1	77	106	14	RAEB-2	45,XY,-5,-7,+8, - 12, - 16,- 22, + marE[?del(11)(q11)], + marC[?der(11)t(?;11;22)], + del(22)(q11)	AraC+Mitho-xanthrone+ Tenoposide	(died) Septicemia 5 months after diagnosis.		[8]
10	64/M	6.9	7.8	98	69	16	RAEB-2	46,XY[7]/47,XY, +8,t(9;22)(q34;q11)[6]	Hydroxyurea	(died)	9	[8]

^*∗*^WBC = white blood cell count; AMC^*∗∗*^ = absolute monocyte count; WHO^*∗∗∗*^ = World Health Organization; ^±^outcome reported at the time of publication; AML^*∗∗∗∗*^ acute myelogenous leukemia; ^∧^BSC = best supportive care; ^++^TKI = tyrosine kinase inhibitor; ^*ε*^patient was diagnosed in April 2001. He received Imatinib for 2 months until August 2001. Given AML transformation, BMT was performed and remained alive by the time of publication in June 2003; ^+^RCMD = refractory cytopenia multilineage dysplasia; ^+++^RCUD = refractory cytopenia multilineage dysplasia; ^*Υ*^RAEB = refractory anemia excess of blast.
